# Outcomes of Open Radical Nephrectomy for Advanced Renal Malignant Tumors in the Minimally Invasive Era: Findings From a Single Institution Over 17 Years

**DOI:** 10.7759/cureus.89860

**Published:** 2025-08-12

**Authors:** Mahmoud Mohammed, Hesham M Hamza, Ali Zedan, Khalid M Rezk, Adel A Makar

**Affiliations:** 1 Surgical Oncology, South Egypt Cancer Institute, Assiut University, Assiut, EGY; 2 Urology, Worcestershire Acute Hospitals NHS Trust, Worcester, GBR; 3 Urology, South Egypt Cancer Institute, Assiut University, Assiut, EGY

**Keywords:** cytoreductive, inferior vena cava thrombus, lymph node metastasis, open radical nephrectomy, preoperative embolization, renal cell carcinoma, surgical outcomes

## Abstract

Background and objective

There has been a growing shift toward minimally invasive surgical approaches, including laparoscopic and robotic techniques, in the management of renal tumors. However, many centers are still performing open surgery for large or complex tumors, particularly those with nodal or vascular invasion, due to concerns about oncologic adequacy and technical limitations. This cohort study discusses the experience at a single institution over 17 years with cases where the urology multidisciplinary team (MDT) reached a consensus to proceed with open surgery electively for optimal oncologic control, especially in patients with high-risk or anatomically challenging disease.

Methods

A retrospective review was conducted of 105 patients who underwent open radical nephrectomy (ORN; including nephroureterectomy) with either curative or cytoreductive intent between January 2007 and June 2024. Data collected included tumor size, clinical stage, nodal status, venous involvement, perioperative outcomes, disease-specific survival (DSS), and overall survival (OS). Preoperative intervention entailing renal arterial embolization was also recorded.

Results

The median tumor size was 10.7 cm, with 69 patients (66%) having tumors ≥10 cm. Locally advanced disease (T3-T4) was present in the majority of cases. Inferior vena cava (IVC) tumor thrombus was identified in 21 patients (20%), while 47 patients (45%) had renal vein thrombus. Clinically positive lymph nodes (cN1) were seen in 40 patients (38%), with pathological confirmation available in a subset. Lymphadenectomy was performed in 51 patients (49%), and IVC thrombectomy in 22 patients (21%). Preoperative embolization was used in 14 patients (13.3%) to facilitate vascular control. No patients received neoadjuvant systemic therapy. Major complications (Clavien-Dindo grade ≥III) were observed in 4% of patients, with 30-day mortality in three cases. Survival outcomes were assessed using Kaplan-Meier analysis.

Conclusions

ORN continues to be performed for large, node-positive renal tumors and those with vascular-invasive features. Despite the advances in minimally invasive surgery (MIS), open surgery continues to offer critical advantages in exposure, vascular control, and complete tumor resection. Surgical proficiency in open techniques must be maintained within a multidisciplinary urologic oncology practice.

## Introduction

The management of renal tumors has witnessed a growing shift toward minimally invasive surgical approaches, including laparoscopic and robotic techniques [1,2]. Nonetheless, open radical nephrectomy (ORN) continues to play a critical role in the treatment of large, complex, or anatomically challenging tumors, where complete resection and vascular control are essential. Many institutions remain cautious in fully adopting these approaches for tumors involving major vasculature or regional lymph nodes, where oncological clearance and technical feasibility remain critical challenges [3]. In this context, ORN continues to hold an essential role in the surgical management of renal tumors with adverse features. At our center, ORN has been selectively employed for cases with anticipated difficulty due to tumor size, venous involvement, or nodal metastases, prioritizing complete resection and oncologic control while balancing surgical risks.

This study aimed to evaluate the perioperative and oncologic outcomes of ORN in patients with advanced renal malignancies, including those with large tumors, nodal involvement, and vascular invasion. While open surgery remains the standard for anatomically complex cases, recent literature lacks large-scale, long-term data detailing the real-world surgical management and outcomes in such patients in the current minimally invasive era. This retrospective longitudinal cohort study draws on 17 years of institutional experience to characterize patient selection, operative strategies, complication rates, and survival outcomes. By doing so, it seeks to reaffirm the role of open surgery in high-risk scenarios and inform future surgical decision-making in multidisciplinary renal cancer care.

## Materials and methods

Ethical considerations

This retrospective study was conducted at Worcestershire Acute Hospitals NHS Trust and was registered with the institutional clinical audit and governance department as a service evaluation (Audit ID: 11942). The study involved analysis of routinely collected, anonymized clinical data and did not involve any intervention or deviation from standard care. All data were anonymized and handled per local research ethics standards.

Study design and patient selection

This was a single-center, retrospective observational study conducted at a single tertiary urology centre (Worcestershire Acute Hospitals NHS Trust, Worcestershire, UK). All patients who underwent ORN or nephroureterectomy with curative or cytoreductive intent between January 2007 and June 2024 were included. Cases were identified through institutional electronic medical records and operative databases.

The inclusion criteria were as follows: patients with a confirmed diagnosis of renal malignancy (e.g., RCC or urothelial carcinoma); those who underwent open radical surgery, either electively or as a planned approach; and no prior nephrectomy or minimally invasive surgery for the index tumor; Patients who underwent minimally invasive (laparoscopic or robotic) or partial nephrectomy were excluded.

Decision regarding surgical approach

The decision to proceed with open surgery was made during multidisciplinary team (MDT) discussions based on preoperative imaging and clinical characteristics. All decisions were made after reaching a consensus agreement in the local MDT. Key factors influencing the choice of open over minimally invasive approaches were as follows: tumor size >10 cm; clinical suspicion of renal vein or IVC thrombus; radiologically positive lymph nodes; anatomic complexity precluding minimally invasive surgery (MIS); and symptomatic presentation requiring rapid decompression or palliative resection

Data collection

The following variables were manually extracted from patient records:

Demographics

Age, sex, and comorbidities.

Tumor Characteristics

Histology, clinical and pathological T/N stage, tumor size, and vascular and nodal involvement.

Surgical Details

Operative time, estimated blood loss, use of lymphadenectomy or IVC thrombectomy, and preoperative embolization.

Perioperative Outcomes

Length of stay, complications graded by Clavien-Dindo, and 30-day mortality.

Oncologic Outcomes

Overall survival (OS) and disease-specific survival (DSS).

Data analysis

Descriptive statistics were used to summarize patient characteristics and outcomes. Continuous variables are presented as medians with ranges, and categorical variables as frequencies and percentages. Survival analyses were performed using the Kaplan-Meier method, with comparisons stratified by surgical intent (curative vs. cytoreductive). All analyses were conducted using EZR software (Saitama Medical Center, Jichi Medical University, Japan), a graphical user interface for R.

## Results

**Figure 1:**
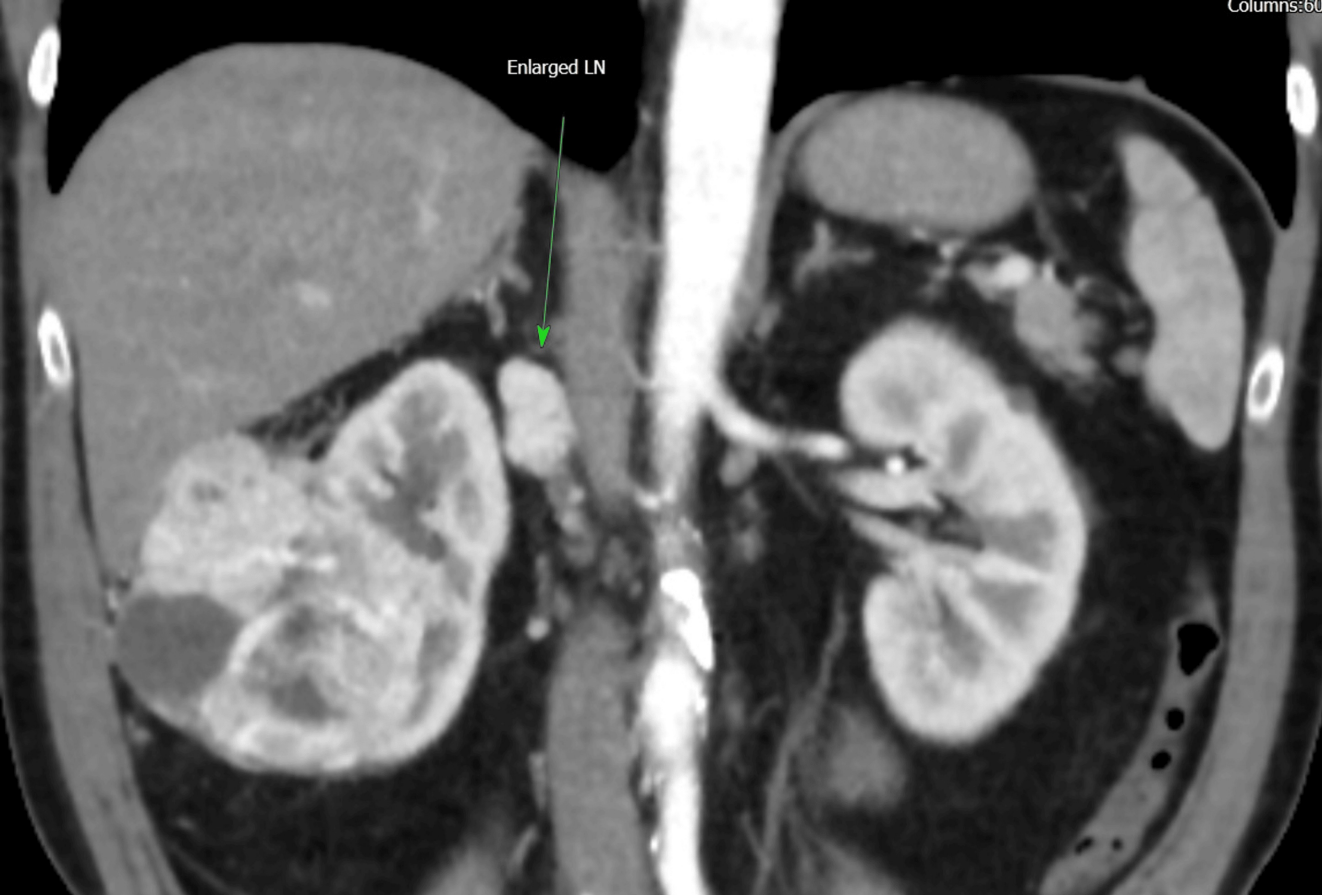
Coronal section of CT scan showing enlarged hilar lymph nodes CT: computed tomography

A total of 105 patients underwent ORN at our institution over the study period. Open surgery was selected based on preoperative features indicating complex disease. The cohort included 66 patients (63%) treated with curative intent and 39 patients (37%) who underwent cytoreductive nephrectomy before systemic therapy. Baseline demographics and tumor characteristics are summarized in Table 1. The median age was 68 years (range: 37-90 years), with males comprising 60% of the population. Clear cell renal cell carcinoma (RCC) was the predominant histology (80%), followed by urothelial carcinoma (10%) and papillary RCC (7%). The median tumor size across all patients was 10.7 cm, with 69 patients (66%) having tumors ≥10 cm. Subgroup analysis revealed that the cytoreductive group had larger tumors (median: 11.6 cm vs. 10.3 cm), a higher rate of tumors ≥10 cm (72% vs. 62%), and a higher frequency of radiologically positive lymph nodes (69% vs. 21%) (Figure 1).

**Table 1:** Patient demographics, tumor characteristics, and surgical intent (n = 105) IVC: inferior vena cava; RCC: renal cell carcinoma

Characteristic	Overall (n = 105)	Curative surgery (n = 66)	Cytoreductive surgery (n = 39)
Age, years, median (range)	68 (37–90)	68 (37–90)	66 (38–87)
Sex – male, n (%)	63 (60%)	37 (56%)	26 (67%)
Sex – female, n (%)	42 (40%)	29 (44%)	13 (33%)
Histology - clear cell RCC, n (%)	84 (80%)	57 (86%)	27 (69%)
Histology - papillary RCC, n (%)	7 (7%)	3 (5%)	4 (10%)
Histology - chromophobe RCC, n (%)	2 (2%)	2 (3%)	0
Histology - collecting duct RCC, n (%)	1 (1%)	1 (2%)	0
Histology - urothelial carcinoma, n (%)	11 (10%)	3 (5%)	8 (21%)
Tumor size, cm, median (range)	10.7 (2.5–26.0)	10.3 (2.5–26.0)	11.6 (4.5–22.0)
Tumor size ≥10 cm, n (%)	69 (66%)	41 (62%)	28 (72%)
Clinical T stage - T1	1	1	0
Clinical T stage - T2	37	25	12
Clinical T stage - T3	53	30	23
Clinical T stage - T3b	1	1	0
Clinical T stage - T4	13	9	4
Clinical N stage - N0	64	52	12
Clinical N stage - N1	41	14	27
IVC tumor thrombus, n (%)	21 (20%)	10 (15%)	11 (28%)
Renal vein thrombus, n (%)	47 (45%)	25 (38%)	22 (56%)
Lymphadenectomy performed, n (%)	51 (49%)	30 (45%)	21 (54%)
IVC thrombectomy performed, n (%)	22 (21%)	12 (18%)	10 (26%)
Pathological nodal disease, n (%)	17/51 (33.3%)	8/30 (26.7%)	9/21 (42.9%)

All patients in the cytoreductive group had metastatic disease (M1), whereas none in the curative group did. Pathologically confirmed nodal metastases were found in 42.9% of cytoreductive cases who underwent lymphadenectomy, compared to 26.7% in the curative group (Table 1). Venous involvement was also more frequent in the cytoreductive group, including inferior vena cava (IVC) tumor thrombus (28% vs. 15%) and renal vein thrombus (56% vs. 38%). Correspondingly, IVC thrombectomy (26% vs. 18%) and lymphadenectomy (54% vs. 45%) were more frequently performed. The discrepancy between radiological IVC thrombus and thrombectomy reflects intraoperative findings, which sometimes necessitated thrombectomy despite a lack of radiological evidence.

None of the patients received neoadjuvant systemic therapy. These findings emphasize the significantly greater disease burden in the cytoreductive group and support the rationale for choosing open surgery in anatomically and biologically complex renal tumors.

Vascular involvement

**Figure 2:**
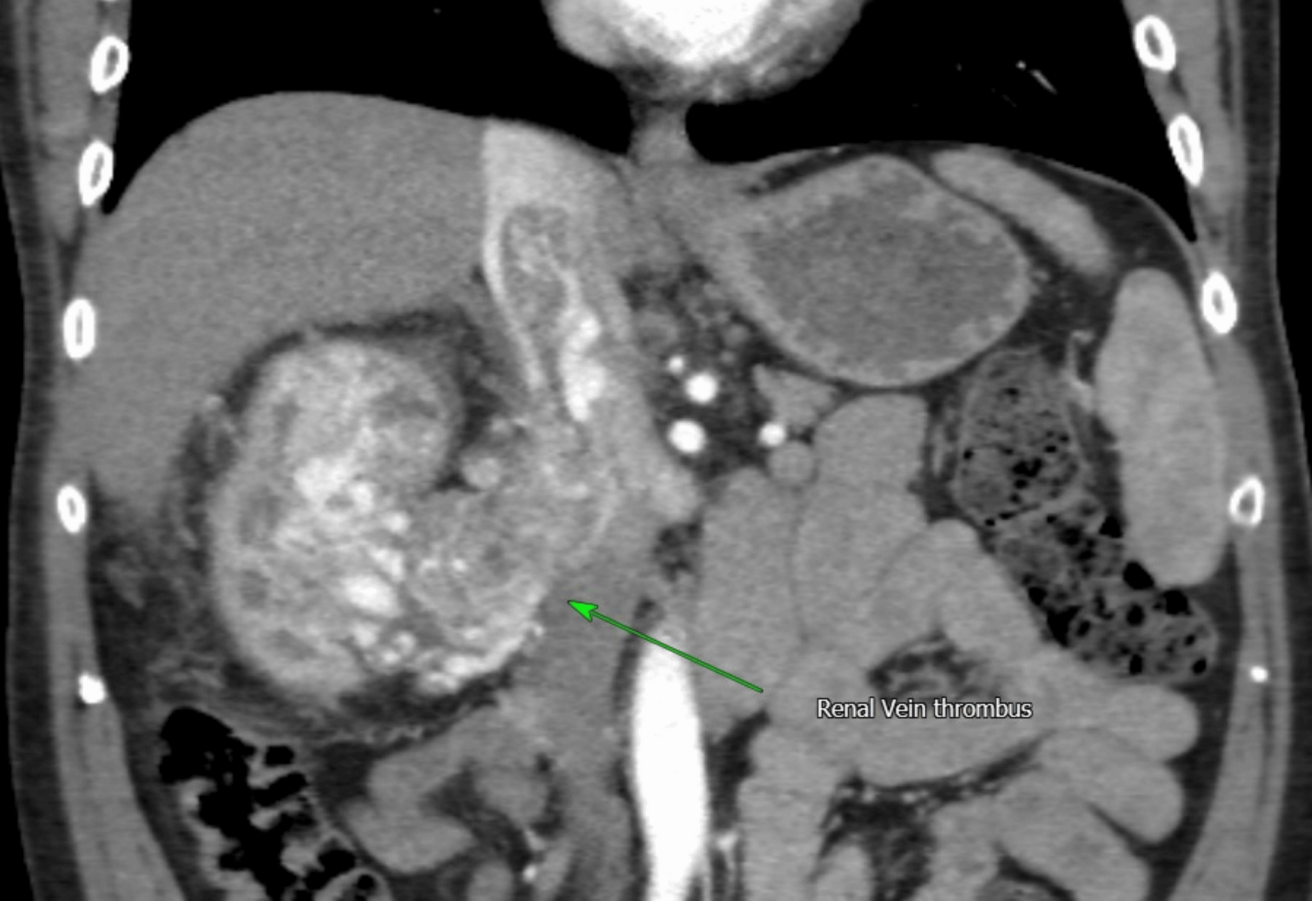
Coronal CT scan showing the level of extent of the IVC thrombus CT: computed tomography; IVC: inferior vena cava

**Figure 3:**
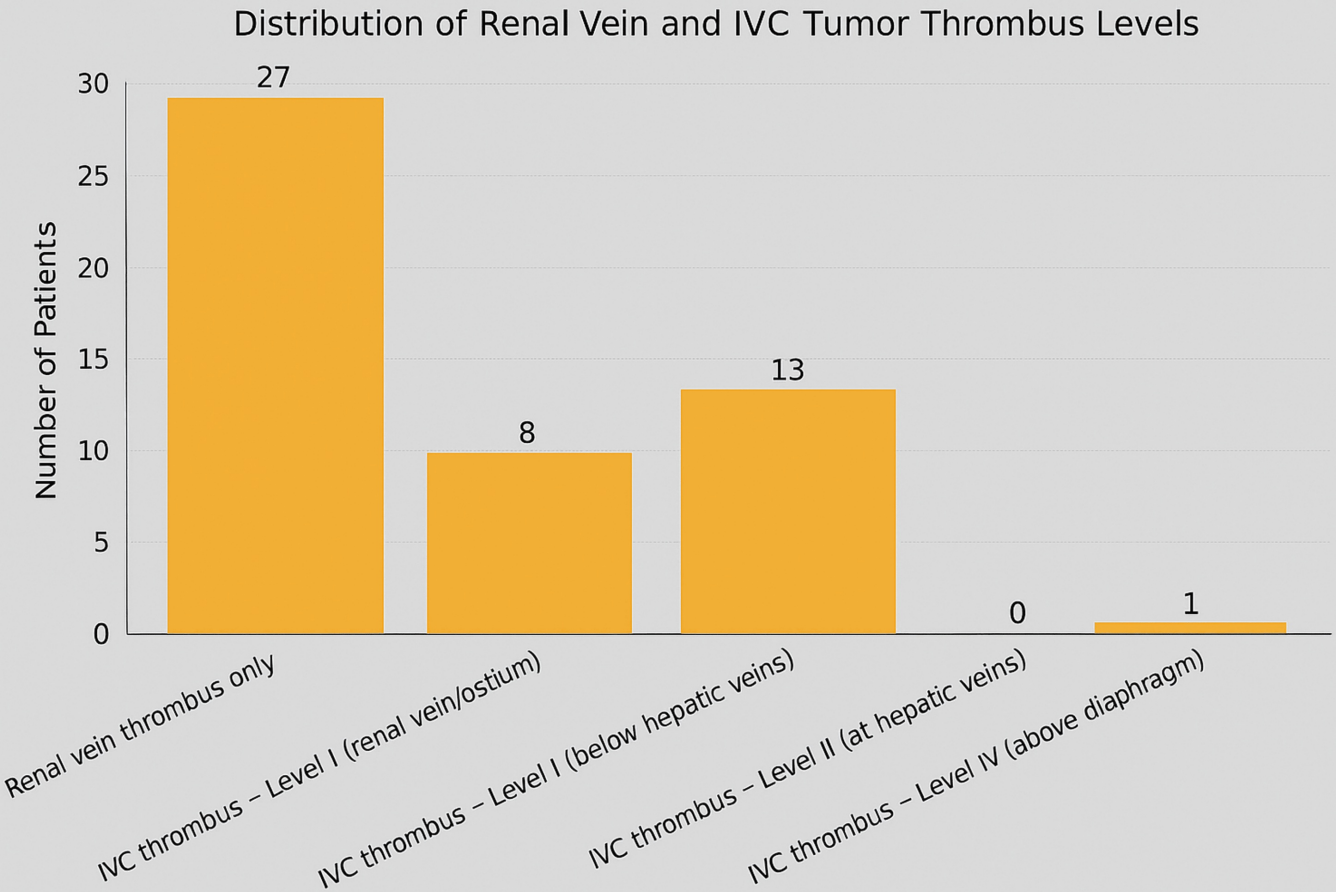
Distribution of renal vein and IVC tumor thrombus levels Renal vein thrombus cases are excluded from the Mayo classification IVC: inferior vena cava

**Figure 4:**
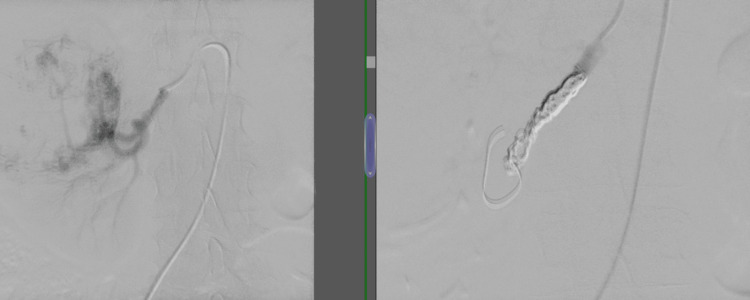
Left: fluoroscopic picture for the renal artery vasculature; right: fluoroscopic confirmation of truncated arterial flow after coil embolization

Tumor thrombus was identified in 27 patients (26%) with renal vein involvement. IVC tumor thrombus was observed in 22 patients (21%), necessitating more advanced surgical management. According to the Mayo classification, eight patients (36%) had Level I thrombus at the renal vein ostium, 13 (59%) had Level II thrombus extending below the hepatic veins, and 1 (5%) had Level IV thrombus extending above the diaphragm. Notably, no patients had Level III thrombus involving the hepatic veins. Renal vein thrombus cases were not classified using the Mayo system. This distribution is illustrated in Figures 2-3. Preoperative renal artery embolization was used in 14 patients (13.3%) to facilitate vascular control (Figure 4).

Surgical management and pathology

All procedures were performed using an open surgical approach. Lymphadenectomy was performed in 51 patients (49%), and IVC thrombectomy was performed in 22 patients (21%). On final pathology, most tumors were staged as pT3, with a smaller proportion being pT4. Among the 51 patients who underwent lymphadenectomy, pathological nodal metastases were confirmed in 33.3% of cases, further supporting the selective use of nodal dissection for patients with suspicious imaging findings (Table 1).

Perioperative outcomes

The mean operative time was 116 minutes, with an average estimated blood loss of 690 mL. Blood transfusions were administered in 16% of patients. Major postoperative complications (Clavien-Dindo grade III or IV) occurred in 1% of cases. Three patients (2.9%) experienced 30-day mortality (Clavien-Dindo grade V), resulting from complications such as empyema, multi-organ failure, or dialysis-dependent renal failure.

Oncologic outcomes

**Figure 5:**
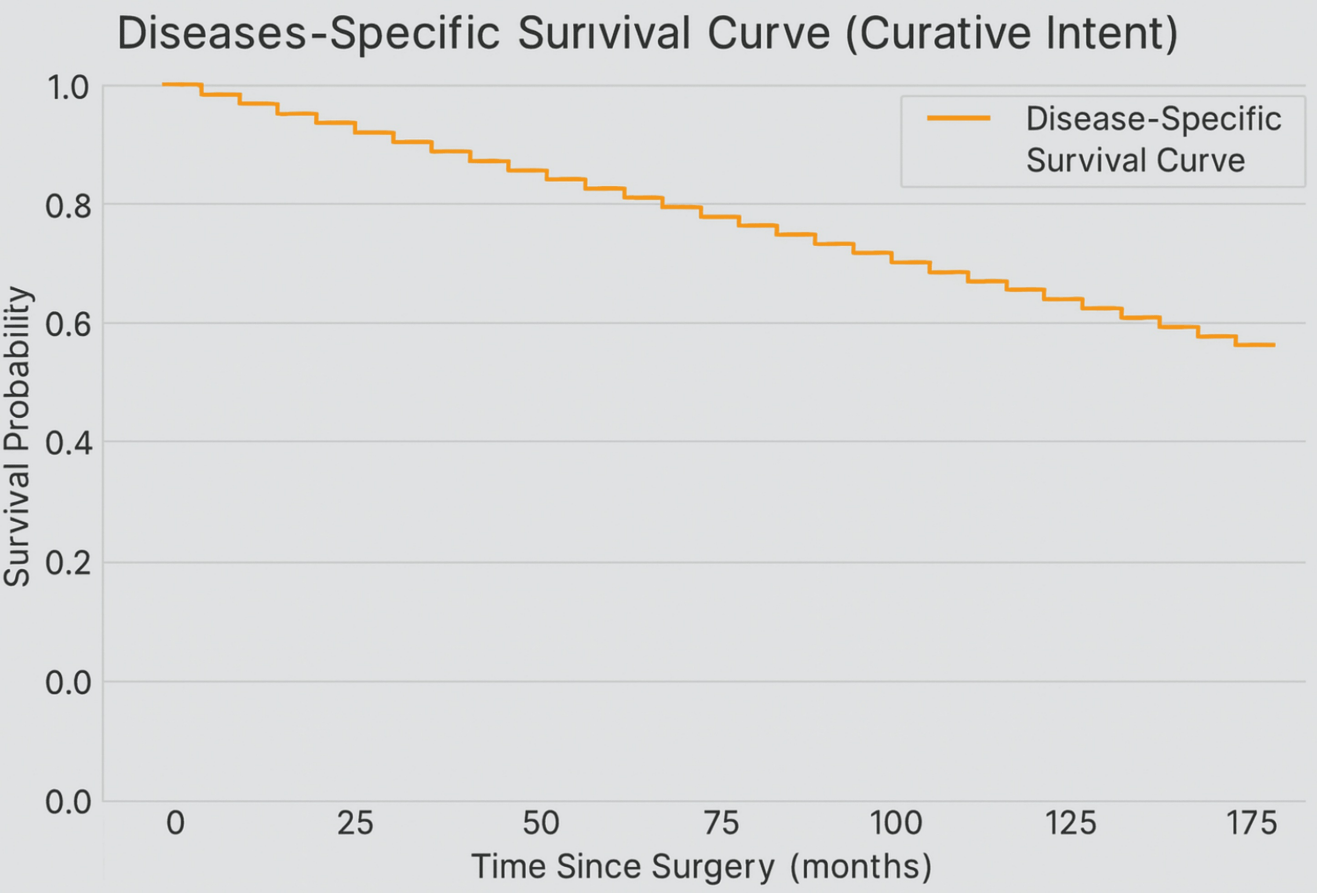
Kaplan-Meier curve showing disease-specific survival for patients undergoing curative-intent surgery Survival remains high in the first 12 months and gradually declines over time. At five years, approximately 65% of patients remain alive without any cancer-related deaths

**Figure 6:**
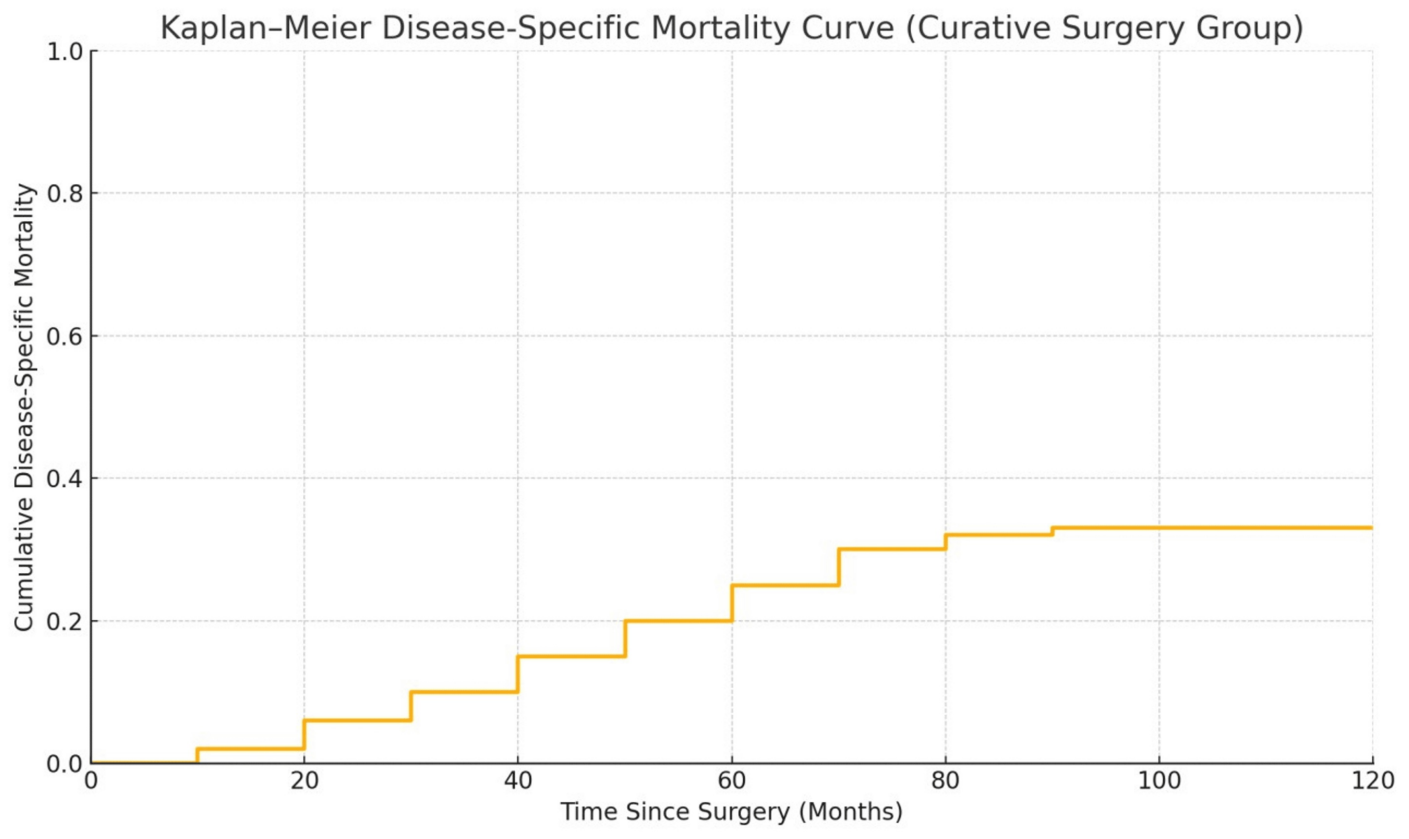
Kaplan-Meier curve showing disease-specific mortality in the curative-intent group Most cancer-related deaths occurred between 12 and 80 months postoperatively. The curve stabilizes beyond this period, reflecting long-term disease control in surviving patients

Survival analysis using the Kaplan-Meier method showed distinct differences based on surgical intent. In the curative group, DSS was favorable: approximately 90% of patients survived at 12 months, and 65% remained alive at five years without cancer-related death (Figure 5). The disease-specific mortality curve indicated that most cancer deaths occurred between 12 and 80 months postoperatively, with the curve stabilizing afterward, suggesting long-term control in survivors (Figure 6). Additionally, 11 patients (16.7%) in this group died from non-cancer-related causes, reinforcing the value of analyzing DSS separately from OS.

**Figure 7:**
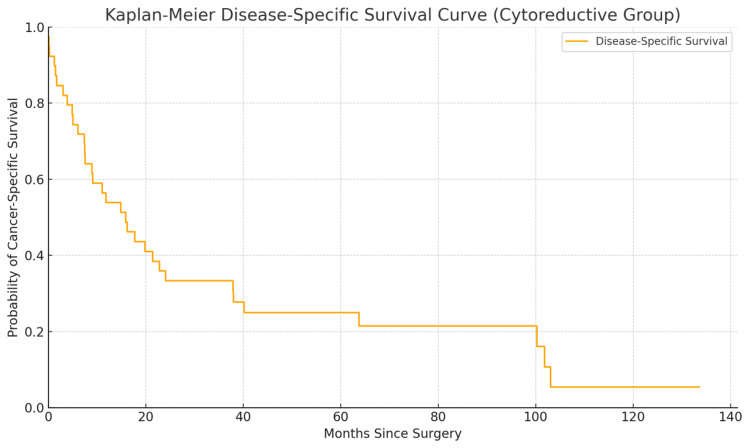
Kaplan-Meier disease-specific survival curve for the cytoreductive group The curve shows a steep decline in survival within the first 36 months following surgery, reflecting the advanced disease burden at presentation. Fewer than 20% of patients remain alive beyond five years

In contrast, the cytoreductive group experienced significantly poorer oncologic outcomes. The DSS curve declined sharply within the first 24-36 months, with fewer than 20% of patients surviving beyond five years (Figure 7). This reflects the aggressive tumor biology and systemic disease burden typical of patients selected for cytoreductive surgery.

These findings demonstrate that open surgery can achieve durable oncologic control in selected high-risk patients with localized disease, while still offering symptomatic relief and a bridge to systemic therapy for patients with metastatic disease.

## Discussion

ORN continues to play a vital role in the management of complex renal tumors, particularly those characterized by large size, nodal involvement, or vascular invasion. While MIS has become the standard of care for small to moderate renal masses, our findings and contemporary evidence reinforce the importance of maintaining proficiency in open surgical techniques for challenging cases. Current European and American guidelines emphasize that ORN remains the preferred approach for large or locally advanced renal tumors, venous tumor thrombus, and cases requiring complex vascular control [2,3].

Tumor size considerations

Large tumor size (≥10 cm) presents unique challenges that often preclude MIS approaches. In our updated cohort, 66% of tumors measured ≥10 cm, with a predominance of locally advanced clinical stage (T3 or higher). Kalapara and Frydenberg [4] emphasize that despite advances in laparoscopic and robotic technologies, ORN remains indispensable for large renal tumors due to these technical and oncologic advantages. MIS for such large masses is associated with longer operative times, higher conversion rates, and increased complication risks [5]. Open surgery allows superior exposure, safer vascular dissection, and complete tumor excision. Our perioperative outcomes - mean operative time of 116 minutes, mean estimated blood loss of 690 mL, and a low major complication rate of 1% - endorse the safety and feasibility of open approaches in this high-risk population.

Lymph node involvement

The role of lymph node dissection (LND) in RCC remains a topic of ongoing debate. In this series, 39% of patients had radiologically suspicious nodal disease, and LND was performed in 49% of cases. Pathologically confirmed nodal metastases were identified in 33.3% of patients. While emerging evidence suggests that LND may not confer a survival benefit in metastatic disease [6,7], it remains critical for staging and may guide adjuvant therapies in patients with clinically evident nodal involvement. The 2024 European Association of Urology (EAU) guidelines advocate for LND in selected patients with radiologic lymphadenopathy [2]. These findings underscore the importance of a targeted approach, reserving LND for patients with high-risk features rather than routine application.

Preoperative embolization and neoadjuvant therapy

The utilization of preoperative renal arterial embolization in the surgical management of RCC with venous tumor thrombus remains a subject of ongoing debate. The role of preoperative renal artery embolization (PRAE) in the surgical management of RCC with venous tumor thrombus remains a subject of ongoing discussion. In our series, PRAE was selectively employed in 14 patients (13.3%) where preoperative imaging demonstrated complex vascular anatomy, making early access to the renal artery technically challenging. The aim was to facilitate safer vascular dissection and reduce intraoperative blood loss. Recent evidence supports this approach: a 2023 systematic review and meta-analysis reported that PRAE before radical nephrectomy significantly reduces intraoperative blood loss without increasing operative time or complication rates, reinforcing its utility in anatomically complex or high-risk surgical candidates [7].

None of the patients received neoadjuvant systemic therapy before surgery. The role of neoadjuvant therapy in locally advanced RCC is still under investigation, with current studies exploring the potential of systemic treatments to downstage tumors and improve surgical outcomes [8]. However, these approaches remain experimental. As novel targeted and immune-based therapies evolve, the role of preoperative systemic treatment may expand, but further prospective validation is needed. Our findings underscore the need for individualized treatment strategies, considering the complex interplay of surgical and adjunctive interventions in managing advanced RCC.

Vascular invasion and tumor thrombus

Vascular invasion, particularly into the renal vein and IVC, poses substantial technical and oncologic challenges in the surgical management of RCC. In our cohort, 27 patients (26%) demonstrated renal vein thrombus, while 22 patients (21%) exhibited IVC involvement necessitating advanced vascular control. Among those with IVC thrombus, eight patients (36%) had Level I involvement (at the renal vein ostium), 13 (59%) had Level II thrombus (extending below the hepatic veins), and one patient (5%) had Level IV thrombus (extending above the diaphragm). Notably, no cases of Level III thrombus were identified.

These complex vascular presentations necessitated open surgical approaches to ensure safe and complete resection, particularly in the context of extensive venous tumor burden [9,10,11]. All patients requiring IVC thrombectomy achieved macroscopically complete excision without intraoperative embolic events. However, three patients (2.9%) experienced 30-day postoperative mortality, reflecting the inherent risks associated with managing such anatomically and biologically advanced disease.

Our series encompassed a broader spectrum of thrombus levels, including Level IV, and consistently achieved complete resection via open surgery. This reinforces the continued necessity of open approaches for managing extensive vascular invasion, where complete control and direct visualization are paramount.

Multidisciplinary integration

The management of advanced renal tumors, including RCC and urothelial carcinoma, has evolved into a multidisciplinary paradigm that incorporates surgical resection, systemic therapy, and longitudinal surveillance. The patients were generally well and fit to withstand a major surgery, with small-volume disease and symptomatic large tumors. The decision to perform nephroureterectomy cases as a cytoreductive approach was a consensus-based decision involving various specialties as a palliative procedure, and to increase the response of the chemotherapy, which is the main line of treatment. Surgery continues to play a foundational role, particularly in patients with a substantial tumor burden, symptomatic presentation, or localized complications. As highlighted by Ray et al. [11] and further supported by Choueiri and Motzer [12], cytoreductive nephrectomy retains therapeutic value when applied judiciously within the context of multimodal care. Our findings align with this framework, demonstrating that even in patients with locally advanced or anatomically complex disease, aggressive surgical intervention offers meaningful clinical benefit. ORN not only facilitates local disease control and symptom relief but also serves as a critical platform from which systemic therapy can be initiated or escalated.

More recently, we have been offering upfront systemic treatment, especially for those with large volume metastases, unless the primary tumor is causing distressing symptoms; then we will consider cytoreductive surgery at a later stage as per the CARMENA study [12,13].

Oncologic outcomes

Our updated analysis demonstrates a favorable DSS profile among patients undergoing curative-intent ORN. The Kaplan-Meier analysis revealed a five-year DSS rate of approximately 65%, suggesting durable long-term survival in a substantial subset. Importantly, early cancer-specific mortality was minimal, with most deaths occurring between 24 and 60 months postoperatively, highlighting the critical importance of long-term surveillance.

In contrast, patients in the cytoreductive group displayed a more aggressive trajectory, with DSS dropping below 20% by five years. Most cancer-specific deaths occurred within the first 36 months following surgery, reflecting the advanced burden of systemic disease at the time of intervention. Nevertheless, a small proportion of patients achieved long-term survival, underscoring the selective utility of cytoreductive nephrectomy within a multidisciplinary framework. These findings emphasize the prognostic influence of surgical intent. Patients treated with curative surgery, despite harboring large or locally advanced tumors, benefited from complete surgical resection and achieved meaningful long-term cancer control. Conversely, those undergoing cytoreductive procedures faced poorer outcomes, but surgery provided symptomatic relief and a platform for initiating systemic therapy. In selected patients, cytoreductive nephrectomy may still offer clinical benefit by improving performance status, alleviating tumor-related symptoms, and facilitating access to targeted or immune-based treatments.

Our results align with previous reports showing that recurrence rates in high-risk RCC may exceed 50%, and that cytoreductive nephrectomy provides benefit only in carefully selected metastatic cases [12,14,15]. Of note, 11 patients in the curative group died from non-cancer causes, which further supports the value of analyzing disease-specific, rather than overall, survival in this context. While national datasets report five-year cause-specific survival (CSS) exceeding 90% in stage I and approximately 78% in stage III RCC [16,17,18], our cohort represents a high-risk population - including patients with IVC thrombus, nodal metastases, and bulky tumors - where MIS approaches were not feasible or appropriate. These outcomes reinforce the importance of individualized surgical planning and the continued relevance of open surgery in modern urologic oncology.

Strengths and limitations

The primary strengths of this study include the size and focus of the cohort, comprising 105 patients with radiologically or surgically complex renal tumors, and the detailed capture of perioperative and oncologic outcomes. However, it has several inherent limitations, including its retrospective design, the lack of a contemporaneous control group undergoing MIS, and its single-institution design, which may introduce selection and institutional bias and limit the external validity of the findings. Despite these constraints, the data provide valuable insight into real-world surgical decision-making and reinforce the enduring role of open surgical expertise. The findings emphasize the importance of maintaining proficiency in open techniques and advocate for a patient-specific, multidisciplinary approach when managing anatomically complex or high-risk RCC.

## Conclusions

In this 17-year retrospective cohort study, ORN achieved durable oncologic control in selected patients with complex renal tumors, with low major complication rates and clear differences in outcomes between curative and cytoreductive cases. These findings directly support the study objective of evaluating the role of open surgery in anatomically and biologically challenging disease. While our results reinforce the enduring role of ORN for complex renal tumors, future research should focus on prospective studies comparing outcomes between open and minimally invasive approaches in anatomically advanced cases. In addition, the integration of neoadjuvant or perioperative systemic therapies warrants further investigation to optimize sequencing strategies. As the landscape of renal cancer treatment continues to evolve, multidisciplinary collaboration and evidence-driven surgical decision-making will remain paramount.
